# Molecular Characterization of a Recombinant NADC30-like PRRSV Strain with a Novel Gene Deletion Pattern in Nsp2 Gene

**DOI:** 10.3390/vetsci12100983

**Published:** 2025-10-13

**Authors:** Zhengqin Ye, Miaojie Zhang, Lin Yuan, Wenqiang Wang, Zhenbang Zhu, Wei Wen, Kegong Tian, Xiangdong Li

**Affiliations:** 1 Jiangsu Co-Innovation Center for Prevention and Control of Important Animal Infectious Diseases and Zoonoses, College of Veterinary Medicine, Yangzhou University, Yangzhou 225012, China; yezhengqin@126.com (Z.Y.); wqwang@yzu.edu.cn (W.W.); zhuzhb@yzu.edu.cn (Z.Z.); 008634@yzu.edu.cn (W.W.); 2 Beijing Sino-Science Gene Technology Co., Ltd., Beijing 102629, China; zhang.miaojie@sslab.com.cn (M.Z.); yuanlin_1982@163.com (L.Y.); 3 National Research Center for Veterinary Medicine, Luoyang 471003, China; 4 Joint International Research Laboratory of Agriculture and Agri-Product Safety, The Ministry of Education of China, Yangzhou University, Yangzhou 225012, China

**Keywords:** PRRSV, isolation, phylogenetic analysis, Nsp2 deletion pattern, recombination

## Abstract

**Simple Summary:**

Porcine Reproductive and Respiratory Syndrome Virus (PRRSV) continues to pose a significant worldwide challenge for swine production. In this study, we investigated a newly identified strain, HeB2023092, responsible for an outbreak in a pig farm in China. The detailed molecular analysis revealed that this strain is genetically related to NADC30-like PRRSV, but possesses a unique, previously unreported deletion pattern in its Nsp2 gene. Furthermore, we discovered its genetic makeup is a blend of different PRRSV strains, indicating it has undergone complex genetic mixing. The constant evolution makes PRRSV a difficult adversary. By monitoring these novel molecular changes and recombination patterns at viral genome level, we can develop more effective vaccines and control strategies to safeguard pig health.

**Abstract:**

PRRSV poses a persistent global challenge to the swine industry due to its rapid evolution. This study aimed to characterize a novel PRRSV2 strain, HeB2023092, isolated from a suspected outbreak in China in September 2023. We performed virus isolation in porcine alveolar macrophages (PAMs), genome sequencing, phylogenetic analysis, and comprehensive genetic characterization. HeB2023092 replicated effectively in PAMs but not in Marc-145 cells. Phylogenetic analysis consistently grouped it with NADC30-like strains (L1.8). Notably, genomic analysis revealed a unique 41-amino acid deletion (478–518 aa) in Nsp2, in addition to the characteristic 111-amino acid deletion of NADC30-like strains. Significant amino acid variations were also found in the antigenic epitopes and N-glycosylation patterns of GP3 and GP5. Comprehensive recombination analysis identified three distinct recombinant regions, revealing a mosaic genome with a predominant NADC30-like backbone. The backbone incorporated genetic sequences from JXA1-like (L8.7) strains in two regions and from NADC34-like (L1.5) strains in one region. These findings highlight the continuous genetic evolution and complex epidemiology of PRRSV, underscoring the critical need for sustained surveillance and detailed characterization of circulating strains to inform effective control and vaccine development strategies.

## 1. Introduction

PRRS represents a major economic affliction impacting pigs globally, causing billions of dollars in yearly losses for the swine industry [1,2]. This disease primarily causes reproductive failures in breeding sows and respiratory issues in growing pigs [3,4]. PRRS is caused by PRRSV, an RNA virus with a single, positive-sense strand, belonging to the family *Arteriviridae* and genus *Betaarterivirus*. This virus is globally delineated into two primary genotypes: PRRSV1 (European type) and PRRSV2 (North American type). Notably, PRRSV2 is the prevalent genotype observed across Asia, particularly within China [5,6].

At least 11 open reading frames (ORFs) are present in the ~15 kb PRRSV genome. Among these, ORF1a and ORF1b encode large polyproteins pp1a and pp1ab, whose proteolytic processing produces non-structural proteins (Nsp1-12) vital for viral replication, pathogenicity, and immunomodulation [7,8]. ORFs 2-7 encode the structural proteins, including GP2a, GP3, GP4, GP5, the envelope protein (E), the membrane protein (M), and the nucleocapsid protein (N), which form the viral envelope and nucleocapsid [9]. Nsp2 is the largest and most variable non-structural protein, often used for phylogenetic analysis and associated with viral virulence and other complex functions in viral life [9,10]. Discontinuous 30-amino-acid or 131-amino-acid deletions in Nsp2 have been reported to delineate highly pathogenic PRRSV (HP-PRRSV) and NADC30-like PRRSV, respectively. Recently, strains resembling NADC34 have shown a continuous deletion of 100 amino acids in Nsp2 [11]. ORF5, a highly variable gene, is frequently employed in phylogenetic analysis. It encodes GP5, a major envelope glycoprotein and primary target for neutralizing antibodies. GP5 contains several crucial epitopes, including B cell, T cell, primary neutralizing (PNE), decoy, and signal epitopes [12,13]. The N-glycosylation sites on GP5 may contribute to immune evasion by shielding neutralizing antibodies [11]. Moreover, as the most glycosylated PRRSV protein, GP3 features seven highly conserved glycosylation sites [14]. GP3 exhibits high immunogenicity and antigenicity, inducing neutralizing antibodies. It also interacts with CD163, a key PRRSV entry receptor, potentially enhancing infectivity [15,16]. Additionally, GP3-induced antibodies experimentally prevent viral infections, though GP3 is less immunogenic than GP5 [14].

The high mutation rate of its RNA-dependent RNA polymerase, coupled with frequent recombination events, PRRSV shows remarkable genetic diversity and rapid evolution [17]. This genetic flexibility results in the continuous emergence of novel strains with altered pathogenicity, antigenicity, and vaccine efficacy, posing persistent challenges for disease control [18]. In China, PRRSV2 genotype is particularly diverse, including CH-1a-like, VR2332-like, HP-PRRSV-like, QYYZ-like, various NADC30-like and NADC34-like strains, which have caused severe outbreaks across the country [18,19]. Given the dynamic nature of PRRSV evolution, continuous surveillance and detailed genetic characterization of circulating strains are essential for understanding their epidemiology, evaluating the effectiveness of existing vaccines, and guiding the development of novel control strategies.

In this study, we successfully isolated and thoroughly characterized a novel NADC30-like PRRSV strain, designated HeB2023092. We employed virus isolation, complete genome sequencing, phylogenetic analysis, homology comparisons of key viral proteins, and recombination detection to elucidate the genetic features and evolutionary origin of this novel isolate. This study aims to enhance our understanding of the molecular epidemiology and genetic diversity of novel NADC30-like strains. Our findings provide critical insights into the molecular epidemiology of PRRSV in China and underscore the importance of ongoing efforts to monitor the emergence and evolution of PRRSV variants.

## 2. Materials and Methods

### 2.1. Sample Collection and Detection

In September 2023, a suspected PRRSV outbreak occurred in a pig farm in Hebei. This fattening pig farm housed approximately 100 growing pigs that had been previously immunized with a commercial PRRSV modified live vaccine. No PRRSV outbreak had been reported on this farm prior to this event. The affected pigs were around 2 months old, exhibiting clinical symptoms such as cough, asthma, and high fever, with a mortality rate of 10%. Lung tissues were collected with the permission of the animal owners. Tissue samples were ground using a freeze grinder (Jinxin, Shanghai, China), and total RNA was extracted from supernatant using the EasyPure Viral DNA/RNA Kit (TransGen, Beijing, China) and then reverse transcribed using HiScript III SuperMix for qPCR (+gDNA wiper) (Vazyme, Nanjing, China). Finally, the cDNA sample was detected using the Quantitative Real-time Polymerase Chain Reaction (qPCR) with Premix Taq^TM^ Version 2.0 (Takara, Tokyo, Japan) and Primers described in the previous report [20] (Table 1).

### 2.2. Virus Isolation

The remaining suspension was centrifuged, filtered using a 0.22-μm filter, and inoculated onto PAMs and Marc-145 cells for virus isolation. PAMs were grown in RPMI 1640 Media (Gibco, CA, USA) with 10% fetal bovine serum. They were kept at 37 °C in an environment with 5% humidified CO_2_. Marc-145 cells were grown in DMEM (Gibco BRL Co., Ltd. in the USA), also with 10% fetal bovine serum, at the same temperature and CO_2_ conditions. When approximately 80% of virus-infected cells exhibited cytopathic effects (CPE), the virus was harvested by freeze-thawing.

### 2.3. Immunofluorescence Assay (IFA)

Cultures from the third passage were collected and inoculated onto PAMs and Marc-145 cells. The CHR6 strain, which can infect both PAMs and Marc-145 cells as described previously [21], served as the positive control. Uninfected cells were designated as a mock control. PRRSV-infected or mock-infected cells were detected via IFA at 48 hpi, following the methodology described previously [11]. Cells were first fixed for 10 min with paraformaldehyde (Biosharp, Hefei, China), then permeabilized for 15 min with 0.5% TritonX-100 (Solarlabio, Beijing, China) in PBS. Following three PBS washes, the cells were blocked using 3% BSA for 30 min at room temperature. Next, IFA experiments were performed using an anti-PRRSV N (4A5) antibody (MEDIAN, Seoul, Republic of Korea) and an Alexa Fluor 555 goat anti-mouse immunoglobulin G (Cell Signaling Technology, Danvers, MA, USA). The visual data was captured by an inverted fluorescence microscope (U-HGLGPS, OLYMPUS, Tokyo, Japan).

### 2.4. Characterization of In Vitro Growth Properties in PAMs

PAMs were infected with HeB2023092 PRRSV at a multiplicity of infection (MOI) of 1. Supernatants from the infected cell cultures were harvested at 0, 12, 24, 36, 48, 60, and 72 hpi, and viral titers were determined by TCID_50_ assays [22].

### 2.5. Genome Sequencing and Analysis

Total RNA of HeB2023092 (P3) was extracted using TRNzol Universal RNA Reagent (Tiangen, Beijing, China). cDNA was synthesized using HiScript III 1st Strand cDNA Synthesis Kit (Vazyme, Nanjing, China). The genome was sequenced using the Denovo approach at the AZENTA SZ NGS Laboratory (Suzhou, China, File S1).

The whole genome sequence of PRRSV was assembled using online tools available on the Galaxy platform (https://usegalaxy.org, accessed on 20 August 2025). Raw sequencing reads (FASTQ) underwent quality control using FastQC (v0.74). Adapter sequences and low-quality bases (Phred score < 20) were trimmed with Trimmomatic (v0.39) in paired-end mode. High-quality reads were then assembled de novo using metaSPAdes (v4.2.0) with default parameters. Assembly quality, including N50 and total length, was assessed via QUAST (v5.3.0). The consensus Porcine Reproductive and Respiratory Syndrome Virus (PRRSV) genome was identified and extracted based on contig length and sequence similarity to known reference genomes. Multiple sequence alignments were analyzed using MAFFT (v7.520) and DNASTar Lasergene (v18.0) software. Seventy representative PRRSV genomic sequences (Table 2) were extracted from GenBank and used for sequence alignments and phylogenetic analysis. The phylogenetic tree was constructed using the maximum-likelihood (ML) method with IQ-TREE (v3.0.1), using multiple sequences of representative PRRSV available in GenBank. Finally, the amino acid sequences of non-structural proteins and structural proteins were obtained using MEGA11 and aligned with MegAlign Pro 17 of DNASTar Lasergene v18.0 [11]. N-linked glycosylation sites of GP3 and GP5 were analyzed on the website http://www.cbs.dtu.dk/services/NetNGlyc, accessed on 3 September 2025 [11].

### 2.6. Confirmation of Nsp2 Deletion by PCR

Nsp2 sequence of HeB2023092 and a NADC30 strain (2023GD-4, GenBank No: OR269980) isolated in our lab were amplified by polymerase chain reaction (PCR) with the primers listed in Table 1 and subsequently sequenced by AZENTA SZ NGS Laboratory.

### 2.7. Recombination Analysis

Six representative strains without recombinant signals from lineages 1, 3, 5, and 8 were selected as minor parent strains. Specifically, the following strains were chosen: NADC30, JS2021NADC34, QYYZ, JXA1, and CH-1a as described in previous research [19]. Recombinant events were evaluated using the Recombination Detection Program 5 (RDP5), which includes the following methods: RDP (R), Chimaera (C), BootScan (B), 3Seq (T), GENECONV (G), MaxChi (M), and SiScan (S) [23]. A potential recombination event was considered to have occurred when at least six out of the seven detection methods yielded positive results [23]. Recombination events were verified using Simplot software (v3.5.1), within a 500 bp sliding window along genomic alignment (50%) [23]. The positions of the recombination breakpoints were evaluated using MEGA11, along with a phylogenetic analysis. Recombination patterns mapping was performed in the R language (v4.5.1).

### 2.8. Statistical Analyses

Statistical analysis was performed using GraphPad Prism 9 software. Data are presented as mean ± standard deviation (SD). Differences in the growth curve at various time points were analyzed using a Two-way analysis of variance (ANOVA) for repeated measures, followed by Bonferroni’s post hoc test for multiple comparisons. *p* < 0.05 was considered to be significant.

## 3. Results

### 3.1. Virus Isolation and Identification

**Figure 1:**
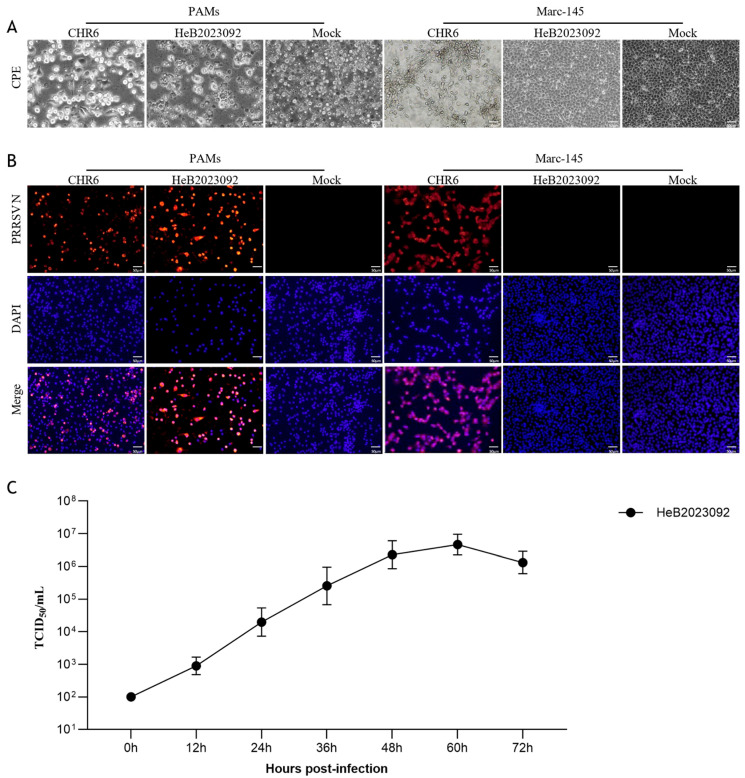
HeB2023092 PRRSV isolation in PAMs and Marc-145 cells. PAMs and Marc-145 cells were either mock-infected or infected with the HeB2023092 strain (P3) or the CHR6 strain for 48 h. (**A**) CPEs were observed using an optical microscope in bright fields. PAMs, scale bar = 200 μm; Marc-145 cells, scale bar = 100 μm. (**B**) Immunofluorescence analysis was conducted using an anti-PRRSV N (4A5) antibody, followed by an Alexa Fluor 555-conjugated anti-mouse IgG (Red) in PAMs, scale bar = 200 μm; Marc-145 cells, scale bar = 100 μm. (**C**) Determination of Viral titers in PAMs. PAMs were infected with HeB2023092 PRRSV at an MOI of 1. The viral supernatants were collected from the infected cells at the indicated time points and titrated by TCID_50_. All tests were performed in triplicate and repeated twice.

We initially detected PRRSV in lung tissue using qPCR, indicating the presence of PRRSV2 (mean Ct = 22.9). The supernatant from the positive tissue was inoculated onto PAMs and Marc-145 cells for virus isolation. Following three passages, the HeB2023092 strain demonstrated successful replication in PAMs, exhibiting noticeable cytopathic effects (CPE) as early as 48 hpi. In contrast, it failed to replicate in Marc-145 cells (Figure 1A). Moreover, IFA results confirmed the presence of PRRSV N protein in HeB2023092-infected PAMs, but not in Marc-145 cells (Figure 1B). These results indicate that the virus was successfully isolated from clinical samples, which replicated in PAMs. Furthermore, viral titers were determined by TCID_50_ at 0, 12, 24, 36, 48, 60, and 72 hpi. Analysis of the one-step growth curve (Figure 1C) revealed an apparent peak in viral titer at 60 h post-infection (hpi) (10^6.0±0.31^ TCID_50_/mL). The analysis indicated that the highest mean titer appeared at 60 hpi. It did not differ significantly from the titers observed at 48 hpi (*p* = 0.15) or 72 hpi (*p* = 0.27). These results suggest that HeB2023092 establishes a plateau phase of high replication efficiency between approximately 48 and 72 hpi.

### 3.2. Genome Sequencing and Phylogenetic Analysis

**Figure 2:**
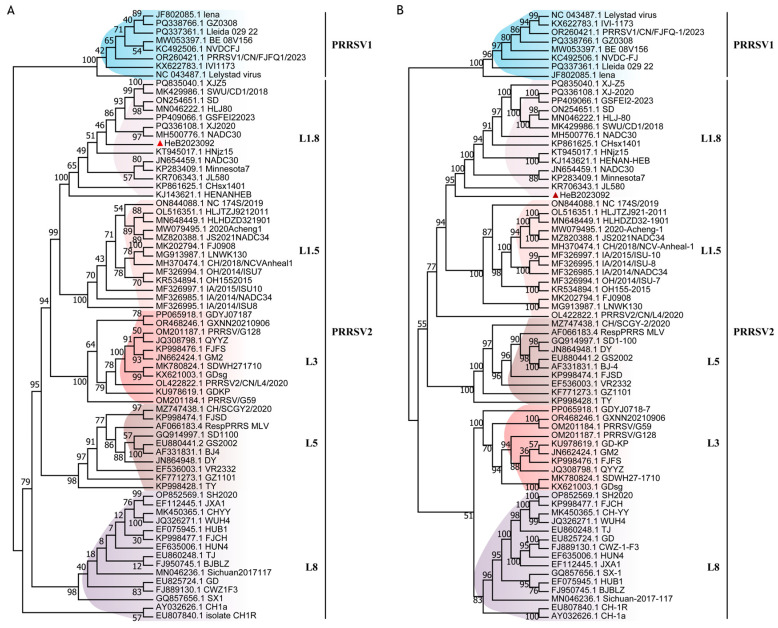
Phylogenetic tree based on the ORF5 gene and whole genome sequence. (**A**) Phylogenetic analysis of HeB2023092 and reference strains based on the ORF5 gene. (**B**) Phylogenetic analysis of HeB2023092 and reference strains based on whole genome sequence. Different lineages are labelled with different color backgrounds. Our isolated HeB2023092 is marked with the red triangle (▲). These trees were generated using the Maximum Likelihood method with IQ-TREE (V3.0.1).

To obtain the whole genome sequence of HeB2023092, the total cDNA of the third passage of HeB2023092 was sequenced on the Illumina platform and assembled. The result showed that the complete genome of HeB2023092 is 14,954-nt in length, excluding the poly (A) tail at the 3′ end, which contains an 188-nt 5′ UTR and a 151-nt 3′ UTR. PRRSV ORF5 gene is known for its variability and is widely used for phylogenetic analysis. To identify the genetic evolutionary relationship between HeB2023092 and 70 representative reference strains, phylogenetic trees were constructed based on both the ORF5 gene and the entire genome. The phylogenetic tree derived from the ORF5 gene demonstrated that HeB2023092 clustered in the same branch as NADC30-like strains (L1.8) (Figure 2A). Similarly, the evolutionary analysis tree on the whole genome also showed that HeB2023092 clustered with NADC30-like strains (L1.8) (Figure 2B).

### 3.3. Homology Analysis

**Figure 3:**
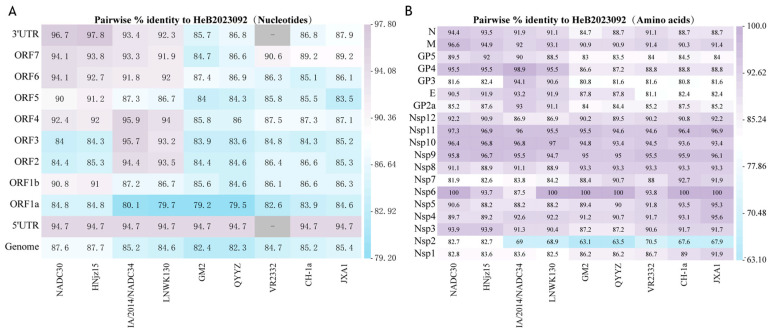
Pairwise distance heatmaps for nucleotides and amino acids. (**A**) Identity of HeB2023092’s entire genome and all ORFs compared to representative reference strains. (**B**) Identity of HeB2023092’s non-structural and structural proteins compared to representative reference strains.

Nine non-recombinant representative PRRSV strains from different lineages were selected to analyze the similarity in nucleotide and amino acid sequence alignments. The results indicated that the HeB2023092 strain had a high whole genome similarity (87.6–87.7%) with L1.8 strains, NADC30, and HNjz15 (Figure 3A). In contrast, the similarities between HeB2023092 and other lineages were relatively low, remaining below 85.4%. Next, the nucleotide homologies of the 5′ UTR, 3′ UTR, ORF1a, ORF1b, and ORF2-7 genes of the HeB2023092 strain were also compared with the reference strains. Most gene segments of HeB2023092 exhibited higher nucleotide homology with NADC30-like PRRSV strains, compared to other strains (Figure 3A), while the ORF1a gene showed similar identity with both NADC30-like strains (84.8%) and JXA1 (84.6%). Additionally, the ORF2-4 gene segments of HeB2023092 exhibited higher nucleotide homology with NADC34-like PRRSV strains (93.2–95.9%) compared to other lineages (Figure 3A).

Furthermore, a comparison of amino acid homologies of viral non-structural proteins and structural proteins between the isolated strain and nine reference strains was performed (Figure 3B). The results indicated that the non-structural proteins Nsp6, Nsp9, Nsp10, and Nsp11 across different lineages demonstrated high similarity. Notably, two non-structural proteins and two structural proteins of HeB2023092 exhibited the highest homology with NADC30-like strains, particularly in Nsp2 (82.7%), Nsp3 (93.9%), M (96.6%/94.9%), and N (94.4%/93.5%) (Figure 3B). Furthermore, four non-structural proteins from the isolated strain showed high homology with L8 strains (CH-1a and JXA1) of PRRSV, particularly in Nsp1 (89.0%/91.9%), Nsp4 (93.1%/95.6%), Nsp5 (93.5%/95.3%), and Nsp7 (92.7%/91.9%) (Figure 3B). Moreover, four structural proteins of the HeB2023092 strain demonstrated high similarity to the IA/2014/NADC34 strain, including GP2a (93.0%), E (93.2%), GP3 (94.1%), and GP4 (98.9%) (Figure 3B). It is also noteworthy that the GP5 protein showed similar identities with both NADC30-like (NADC30/HNjz15) (89.5%/92%) and NADC34-like strains (IA/2014/NADC34/JS2021NADC34) (90.0%/88.5%).

### 3.4. Amino Acid Deletion Analysis in Nsp2

**Figure 4:**
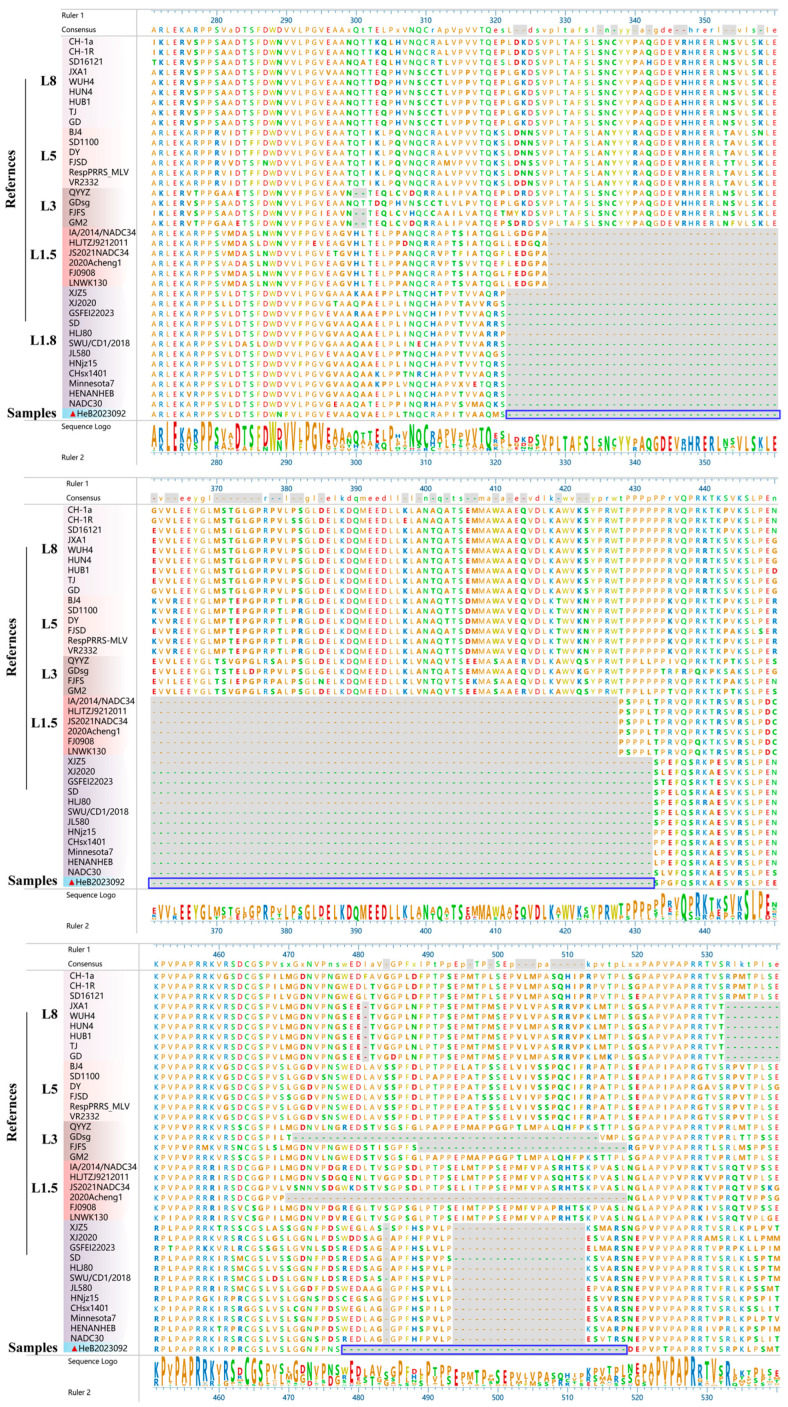
Sequence alignment of Nsp2 amino acids. HeB2023092 strain is highlighted in a red triangle (▲). The two deletion regions of the isolated strain are labelled with blue boxes.

Amino acid alignment revealed that, in comparison to VR2332, HeB2023092 PRRSV displayed a continuous deletion of 111 amino acids (322–432 aa) (Figure 4). This typical pattern of deletion has been characterized in NADC30-like strains. Additionally, this isolated strain shows another continuous deletion of 41 amino acids, occurring between positions 478 and 518 (Figure 4), which has not been reported previously. To further confirm this deletion pattern, the Nsp2 gene was amplified using PCR, with a NADC30-like strain as control. The results showed that the PCR products of the HeB2023092 strain (lane 2) is smaller than those of NADC30-like PRRSV (lane 1) (Figure S1A). Subsequently, the PCR products of Nsp2 from HeB2023092 were sequenced, and the results were consistent with the initial genome sequence assessment (Figure S1B).

### 3.5. Amino Acid Analysis of GP3

**Figure 5:**
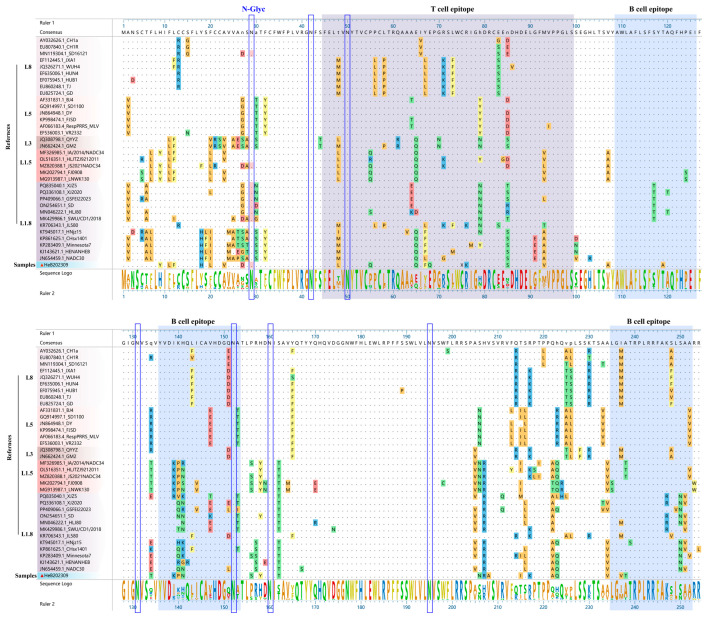
Amino acid sequence alignment of GP3 from the HeB2023092 strain and 37 representative PRRSV strains. T cell antigenic epitope (45–99 aa) highlighted with a purple background. Three previously identified B-cell epitopes (109–126 aa, 136–153 aa, and 235–252 aa) are highlighted with a blue background, respectively. Predicted N-glycosylation sites are indicated by blue boxes, with inactive sites further distinguished by a pink background.

Previous studies have identified one T cell antigenic epitope (45–99 aa) and three critical B cell antigenic epitopes (109–126 aa, 136–153 aa, and 235–252 aa) within the GP3 protein [14]. Notably, the HeB2023092 strain showed distinct amino acid variations compared to the NADC30 strain within these regions (Figure 5). Specifically, seven amino acid differences were found in the T cell epitope (positions 55, 57, 67, 68, 71, 76, 91). For the B cell epitopes, one difference was noted in the first epitope (109–126 aa, position 119), two in the second (136–153 aa, positions 139, 140), and four in the third (235–252 aa, positions 237, 238, 250, 252). Furthermore, the N-glycosylation site at position 29 in the HeB2023092 strain was below the detection threshold (<0.5), a finding also consistently observed in seven other strains, including one strain in L8 (SD1612-1), one strain in L1.5 (JS2021NADC34), and five strains in L1.8 (Figure 5).

### 3.6. Amino Acid Analysis of GP5

**Figure 6:**
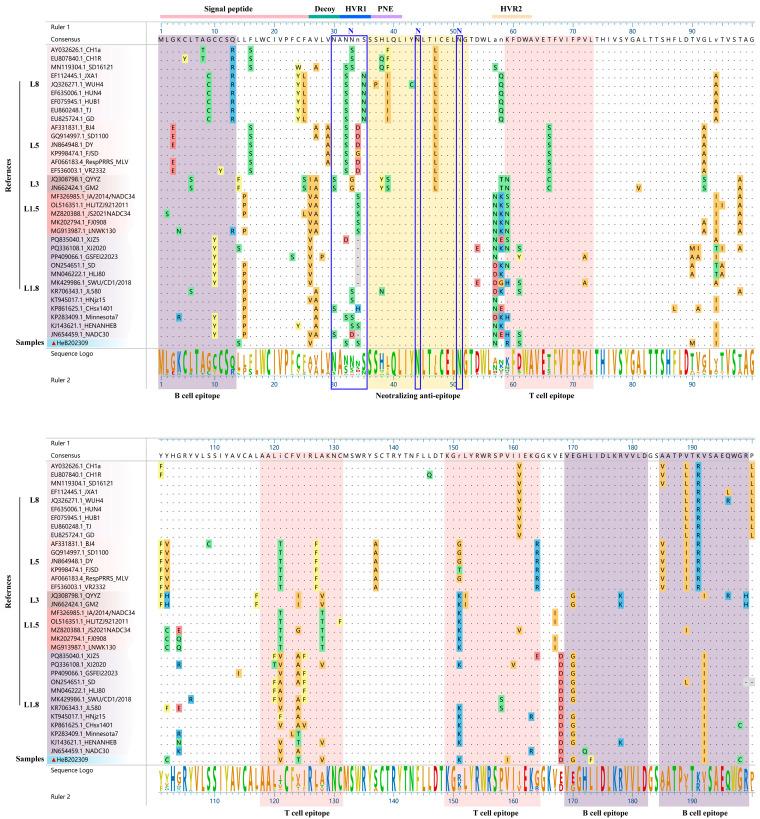
Alignment of GP5 amino acid sequences. The isolated HeB2023092 strain is highlighted with a red triangle (▲). Key functional regions are indicated by distinct background colors: the neutralizing epitope (yellow), three T-cell antigenic regions (pink), and three B-cell antigenic regions (purple).

As depicted in Figure 6, comparative analysis with NADC30 strain revealed several amino acid mutations in HeB2023092 strain. Specifically, three mutations (Y10C, L14S, and P15L) were identified within the signal peptide region. Additionally, two mutations (A27V and S30N) were observed in the decoy epitope region. Furthermore, HeB2023092 strain exhibited amino acid mutations in two T cell epitopes: A124T and A128V in T cell epitope (118–131 aa), and R151K, V159I, and R163K in T cell epitope (149–164 aa). Five amino acid mutations were also observed across the B cell epitopes: Y10C in B cell epitope (1–13 aa), E170G, Q172H, and L173F in B cell epitope (169–182 aa), and G198C in B cell epitope (185–199 aa).

By utilizing an online tool, we predicted the N-glycosylation sites of GP5 across various PRRSV strains. Notably, the HeB2023092 strain was found to possess four potential N-glycosylation sites (N30, N33, N44, and N51). This pattern was consistent with that observed in most Chinese VR2332-like strains (BJ-4, sd1-100, ResPRRS MLV, and DY) and QYYZ-like strains (FJSD and QYYZ) (Table 3).

### 3.7. Identification and Characterization of Recombinant Events and Genomic Regions

**Figure 7:**
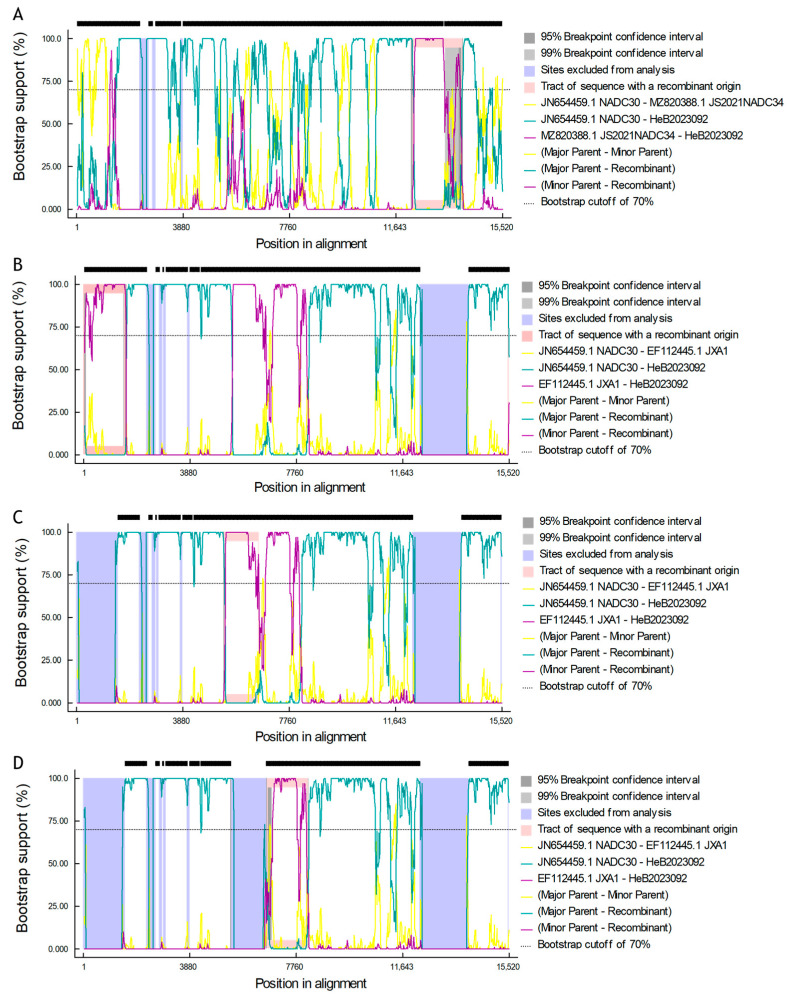
Bootscan analysis of the HeB2023092 strain. (**A**) Recombinant Event 1. Major strain NADC30 as primary parent; minor strain JS2021NADC34 as contributor. (**B**) Recombinant Event 2. Major strain NADC30 as parent; minor strain JXA1 as contributor. (**C**) Recombinant Event 3. Major strain NADC30 as parent; minor strain JXA1 as contributor. (**D**) Recombinant Event 4. Major strain NADC30 as parent; minor strain JXA1 as contributor. This Bootscan plot, generated by RDP5, provides evidence for recombination within the full-genome sequence of the HeB2023092 strain. The *X*-axis represents the nucleotide position, while the *Y*-axis indicates the bootstrap support value (%).

For gene recombination analysis, we used the complete genome of the HeB2023092 strain and six representative strains from China, including VR2332, CH-1a, JXA1, QYYZ, NADC30, and JS2021NADC34. Recombinant events were initially determined using RDP5. A comprehensive recombination analysis conducted with RDP5 revealed four distinct recombination events within the dataset. Each event was strongly supported by a consensus of all seven detection methods (RDP, GENECONV, BootScan, MaxChi, Chimaera, SiScan, and 3Seq). The details of these recombination events, including the recombinant strain, putative parental sequences, approximate breakpoint positions, and supporting statistical values, are summarized in Table 4 and illustrated by Bootscan analysis in Figure 7. The results indicated that the major parental strain for the four recombinant events was NADC30 PRRSV. The first recombinant event occurred at the breakpoint positions between nt12,152 and nt13,961, with NADC34 serving as the minor parental strain. The other three recombinant events were identified at the following breakpoint positions: nt1–1508, nt5280–6568, and nt6589–8076, with JXA1 as the minor parental strain.

**Figure 8:**
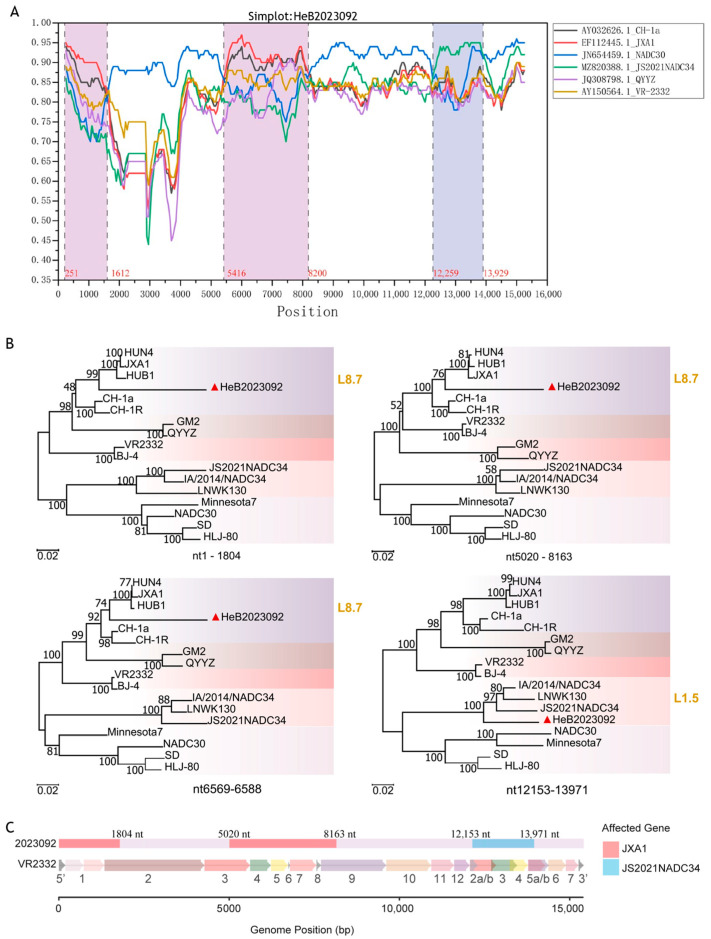
Recombination and phylogenetic analysis of recombinant segments. (**A**) Simplot analysis of recombination events. This panel illustrates the recombination analysis of the HeB2023092 strain, performed using Simplot software (v3.5.1). Different colors represent various reference strains, and distinct recombinant regions are highlighted with corresponding-colored backgrounds. (**B**) Phylogenetic trees for recombinant segments. Phylogenetic trees were constructed based on the three identified recombinant regions. The isolated HeB2023092 strain is highlighted with a red triangle (▲). These trees were generated using the Maximum Likelihood method with IQ-TREE (v3.0.1). (**C**) Genomic mapping of recombination patterns. This panel presents a visualization of recombination patterns, generated in the R language (v4.5.1), superimposed on the full-length genome structure (referenced to VR-2332). It delineates the positions and boundaries of major ORFs.

Simplot analysis revealed three major recombination regions, a finding further supported by subsequent phylogenetic analysis of these regions (Figure 8A,B). Notably, two adjacent events detected by RDP5, specifically nt5280–6568 and nt6589–8076, appeared merged into a single continuous region in the Simplot results. Phylogenetic analysis of the intervening segment (nt6569–6588) confirmed its high sequence similarity and shared ancestry with L8.7 (JXA1-like) strains (Figure 8B). The final breakpoint positions were accurately determined through alignment with MEGA11 and visualized using the R language(v4.5.1). Specifically, the first interval (nt1-1804) encompasses the 5′UTR, Nsp1, and the C-terminal region of Nsp2 (Figure 8C). The second interval (nt5020–8163) corresponds to the N-terminal region of Nsp3 through to the C-terminal region of Nsp9 (Figure 8C). The third interval (nt12,153–13,971) is located in ORF2-4 and the C-terminal region of ORF5 (Figure 8C).

## 4. Discussion

PRRSV continues to pose a significant threat to the global swine industry, necessitating continuous monitoring and characterization of emerging strains. The PRRSV2 is circulating in China and can mainly be classified into four lineages (L1.8, L1.5, L3.5, and L8). L1.8 (NADC30-like) has been the predominant strain in China since 2016. Additionally, there is an increasing proportion of recombinant strains associated with NADC30 [18].

The PRRSV outbreak investigated in this study occurred on a pig farm that had previously immunized its pigs with a commercial PRRSV modified live vaccine. Despite prior vaccination, the farm experienced clinical symptoms of PRRSV in 2-month-old pigs, including cough, asthma, and high fever, with a 10% mortality rate. This observation highlights the potential for vaccine escape by the emerging PRRSV strains and underscores the inadequacy of current commercial vaccines in providing complete protection against novel variants, particularly NADC30-like strains, which aligns with previous findings [23]. We successfully isolated a novel PRRSV strain in PAMs, which are well-established for PRRSV isolation and propagation [24]. However, this isolated strain demonstrated an inability to infect Marc-145 cells. This cellular tropism pattern is frequently observed in NADC34-like and some NADC30-like strains [25]. Phylogenetic analysis, consistently supported by both ORF5 and whole-genome sequences, firmly clustered HeB2023092 within the NADC30-like strains of L1.8. This genetic background is highly relevant given the widespread circulation and significant clinical impact of NADC30-like PRRSV in China.

Further comprehensive homology analysis largely corroborated these phylogenetic relationships, demonstrating the highest nucleotide and amino acid similarities of HeB2023092 with L1.8 strains. However, an intriguing mosaic pattern of genetic relatedness emerged across different genomic segments and proteins. While most non-structural proteins (Nsp2, Nsp3, Nsp10, Nsp11, Nsp12) exhibited the highest homology with NADC30, other non-structural proteins (Nsp1, Nsp4, Nsp5, Nsp7) showed higher similarity to L8 strains (CH-1a and JXA1). More strikingly, most structural proteins, including GP2a, E, GP3, GP4, and the C-terminal region of GP5, displayed higher homology with L1.5 strains, specifically IA/2014/NADC34. This initial indication of a mosaic genome strongly suggested the occurrence of complex recombination events in the evolutionary history of HeB2023092.

The Nsp2 gene is recognized for its highest genetic diversity, and its protease activity is crucial in viral replication and host immunity modulation [10]. Alignment of the HeB2023092 Nsp2 amino acid sequence with representative reference strains revealed a characteristic 111-amino acid deletion (322–432 aa), consistent with NADC30-like strains. Intriguingly, this isolated strain presented an additional and previously unreported continuous 41-amino acid deletion (478–518 aa) (Figure 4). The 41 amino acid sequence is situated in a region previously studied by Han et al. They found that the amino acid region from 324 to 523 of Nsp2 is not essential for replication in Marc-145 cells [26]. However, potential mutations in Nsp2 itself could also influence this tropism, as Nsp2 has been implicated in altering the cellular environment [10]. Thus, the specific influence of this novel deletion in the isolated strain on cell tropism requires further investigation. Moreover, Nsp2 has been identified as containing the highest frequency of immunogenic epitopes. Among these, 18 were found to be immunoreactive with over 50% of the tested sera, with ten of these peptides reacting with 80–100% of the examined sera [27]. Our analysis revealed that the deletion region of Nsp2 in the HeB2023092 strain encompasses two reactive B cell epitopes (^476^PDGREDLTVGGPLDL^490^ and ^496^PMTPLSEPALMPALQ^510^) [27]. Beyond immune escape via epitope loss, it is plausible that this deleted region, potentially overlapping with other uncharacterized functional domains, might influence Nsp2′s roles in viral replication efficiency or its antagonism of host immune responses, warranting further investigation. Such substantial deletions in Nsp2 represent a critical distinguishing feature of HeB2023092, warranting further functional studies. Collectively, these results highlight the emergence of this novel PRRSV strain and underscore the genetic characteristics facilitating immune escape in circulating PRRSV variants [28].

GP3, an important structural protein, contains one T cell epitope and three B cell antigenic epitopes [14]. We observed a higher frequency of mutations within the T cell antigenic epitope of HeB2023092, consistent with its designation as a highly variable region (HVR) [14]. Conversely, the amino acid sequence within the B cell epitopes demonstrates relative conservation. These observed differences in antigenic epitopes and the potential alterations in the secondary structures of GP3 sequences across various typologies and strains may underline this phenomenon. Glycosylation, particularly N-glycosylation, is pivotal for protein folding [29], immune evasion [30], and epitope recognition [31], underscoring its relevance in understanding PRRSV strain dominance. Regarding N-glycosylation, the site at amino acid position 29 (N29) in HeB2023092 was predicted to be below a critical threshold (<0.5), indicating reduced or absent glycosylation. This finding was consistently observed in seven other strains across L8, L1.5, and L1.8 lineages. Such variations, including reported mutations like N29D in strains like GD20220303, underscore how GP3 glycosylation extent significantly influences viral immune evasion [15]. Consequently, these amino acid changes and glycosylation patterns in GP3 could profoundly alter the virus’s antigenicity, impacting vaccine efficacy and diagnostic tool development [14].

GP5 serves as a major envelope protein and the primary target for neutralizing antibodies [15]. Our analysis revealed HeB2023092 GP5 exhibits similar amino acid homology with both L1.8 and L1.5 strains. We identified several mutations in critical GP5 epitopes when compared to NADC30 and other reference strains. These included three mutations (Y10C, L14S, P15L) in the signal peptide region, and two mutations (A27V, S30N) in the decoy epitope region. Additionally, multiple mutations were present in T cell epitopes (A124T, A128V, R151K, V159I, R163K) and B cell epitopes (Y10C, E170G, Q172H, L173F, G198C). Such alterations in crucial antigenic regions can influence the binding of neutralizing antibodies and the induction of T cell responses, potentially facilitating immune escape. Mutations in the decoy epitope and primary neutralizing epitope are particularly valuable as markers for studying PRRSV evolutionary relationships in China [32]. The prediction of N-linked glycosylation sites in GP5 is also highly relevant for understanding immune evasion mechanisms [15]. While previous studies identified nine potential N-glycosylation residues with varied combinations over time [33], HeB2023092 uniquely possessed four potential N-glycosylation sites (N30, N33, N44, and N51). This pattern was consistent with most Chinese VR2332-like and QYYZ-like strains (Table 3). This consistency with older VR2332-like strains could represent a reversion to an ancestral glycosylation pattern, or alternatively, convergent evolution where this specific pattern offers a fitness advantage against current host immune responses. This change may result from vaccine-induced immune pressure. Since glycosylation can shield antigenic epitopes from being recognized by antibodies, alterations in glycosylation patterns have important implications for vaccine design and the development of effective antiviral strategies.

The comprehensive recombination analysis, employing both RDP5 and Simplot software, unequivocally identified three distinct recombinant regions within the genome of HeB2023092 (Figure 7 and Figure 8A). These events were strongly supported by multiple detection methods and statistical significance. Specifically, recombination evidence was found in intervals within the 5′UTR/Nsp1/C-terminal Nsp2 (nt1–1804), Nsp3-9 (nt5020–8163), and ORF2-5 (nt12,153–13,971) regions. Phylogenetic analysis of these recombinant segments confirmed contributions from L8.7 (JXA1-like strains) and L1.5 (NADC34-like strains) (Figure 8C), integrated into an overall NADC30-like backbone. This robust evidence of recombination, particularly among NADC30-like, JXA1-like, and NADC34-like strains, represents a critical finding. This mosaic genome suggests a potential evolutionary advantage, where HeB2023092 may have acquired enhanced replication or immune modulation features from JXA1-like non-structural proteins, while simultaneously adopting specific cell entry or antigenic characteristics from NADC34-like structural proteins, creating a highly adapted and divergent strain. This unique combination of recombination events, together with the novel Nsp2 deletion, highlights the ongoing complex and dynamic evolutionary landscape of PRRSV and underscores the importance of understanding novel recombination events for effective disease control and vaccine development. The observed recombination region on ORF2-5, with its NADC34-like origin, involves genetic contributions to minor envelope proteins (GP2a–GP3). These proteins are known to influence cell tropism [25], which might partially explain the specific cell tropism of HeB2023092 for PAMs and its inability to infect Marc-145 cells. The clinical observations from the affected pig farm, characterized by respiratory symptoms and a 10% mortality rate in previously vaccinated 2-month-old pigs, further emphasize the virulence of this novel HeB2023092 strain. The high mortality despite prior immunization points towards a significant immune escape mechanism, likely driven by the identified genetic alterations, including the unique Nsp2 deletion and the mosaic genome architecture resulting from recombination events. This scenario highlights the urgent need for updated vaccine strategies and continuous surveillance to combat the evolving nature of PRRSV, especially in regions with vaccination programs.

In conclusion, we thoroughly characterized a novel PRRSV2 strain—HeB2023092, isolated from an outbreak in 2023 in Hebei, China. Phylogenetic and homology analyses confirmed its clustering within the L1.8 NADC30-like lineage, a predominant strain in China. Significantly, HeB2023092 displayed a unique 41-amino acid deletion (478–518aa) in Nsp2, encompassing key B-cell epitopes, alongside characteristic NADC30-like deletions. Mutations in epitopes and altered glycosylation patterns in GP3 and GP5 proteins further highlight their antigenic distinctiveness. Moreover, the strain exhibited a mosaic genome, resulting from three distinct recombination events between NADC30-like, JXA1-like (L8.7), and NADC34-like (L1.5) strains. The clinical symptoms and mortality observed in vaccinated pigs on the farm underscore the epidemiological relevance and potential for severe impact of this novel strain, necessitating further investigation into its pathogenicity. Future research will focus on in vivo studies, including challenge experiments on piglets and pregnant sows to evaluate its clinical morbidity, virulence, and pathogenicity. Importantly, the unique deletion patterns in Nsp2 and their functional impact on virulence and pathogenicity can be further elucidated through the construction of infectious clones.

## Figures and Tables

**Table 1:** The primer sequences used in this study.

Primer	Primer Sequences (5′-3′)
PRRSV2-UF	TTGTGCTTGCTAGGCCGC
PRRSV2-UR	ACGACAAATGCGTGGTTATCA
PRRSV-UP	FAM-TCTGGCCCCTGCCCA-MGB
Nsp2-F	GCTGGAAAGAGAGCAARAAAAAC
Nsp2-R	GCCCAGTAACCTGCCAAGAAC

**Table 2:** Seventy representative PRRSV strains used in this study.

No.	Strains	GenBank Accession No	Origin	No	Strains	GenBank Accession No	Origin
1	SD	ON254651.1	China	36	PRRSV/G59	OM201184.1	China
2	HLJ-80	MN046222.1	China	37	SH2020	OP852569.1	China
3	SWU/CD1/2018	MK429986.1	China	38	JXA1	EF112445.1	China
4	XJ-Z5	PQ835040.1	China	39	CH-YY	MK450365.1	China
5	GSFEI2-2023	PP409066.1	China	40	WUH4	JQ326271.1	China
6	XJ-2020	PQ336108.1	China	41	Sichuan-2017-117	MN046236.1	China
7	NADC30	JN654459.1	China	42	HUB1	EF075945.1	China
8	HNjz15	KT945017.1	China	43	FJCH	KP998477.1	China
9	HENAN-HEB	KJ143621.1	China	44	HUN4	EF635006.1	China
10	CHsx1401	KP861625.1	China	45	BJBLZ	FJ950745.1	China
11	JL580	KR706343.1	China	46	TJ	EU860248.1	China
12	Minnesota7	KP283409.1	USA	47	GD	EU825724.1	China
13	IA/2014/ISU-8	MF326995.1	USA	48	CWZ-1-F3	FJ889130.1	China
14	IA/2014/NADC34	MF326985.1	USA	49	SX-1	GQ857656.1	China
15	IA/2015/ISU-10	MF326997.1	USA	50	CH-1a	AY032626.1	China
16	NC 174S/2019	ON844088.1	USA	51	CH-1R	EU807840.1	China
17	OH/2014/ISU-7	MF326994.1	USA	52	NADC30	JN654459.1	USA
18	OH155-2015	KR534894.1	USA	53	GS2002	EU880441.2	China
19	CH/2018/NCV-Anheal-1	MH370474.1	China	54	BJ-4	AF331831.1	China
20	HLJTZJ921-2011	OL516351.1	China	55	SD1-100	GQ914997.1	China
21	HLHDZD32-1901	MN648449.1	China	56	RespPRRS MLV	AF066183.4	USA
22	2020-Acheng-1	MW079495.1	China	57	DY	JN864948.1	China
23	FJ0908	MK202794.1	China	58	VR2332	EF536003.1	USA
24	LNWK130	MG913987.1	China	59	FJSD	KP998474.1	China
25	JS2021NADC34	MZ820388.1	China	60	CH/SCGY-2/2020	MZ747438.1	China
26	QYYZ	JQ308798.1	China	61	GZ1101	KF771273.1	China
27	GM2	JN662424.1	China	62	TY	KP998428.1	China
28	FJFS	KP998476.1	China	63	GZ0308	PQ338766.1	China
29	SDWH27-1710	MK780824.1	China	64	NVDC-FJ	KC492506.1	China
30	GDsg	KX621003.1	China	65	BE 08V156	MW053397.1	Belgium
31	PRRSV/G128	OM201187.1	China	66	Lelystad virus	NC_043487.1	The Netherlands
32	GXNN20210906	OR468246.1	China	67	IVI-1173	KX622783.1	Switzerland
33	GD-KP	KU978619.1	China	68	lena	JF802085.1	Belarus
34	PRRSV2/CN/L4/2020	OL422822.1	China	69	Lleida 29 22	PQ337361.1	Spain
35	GDYJ0718-7	PP065918.1	China	70	PRRSV1/CN/FJFQ-1/2023	OR260421.1	China

**Table 3:** Predicted N-glycosylation sites of PRRSV GP5 protein.

Strains	N-Glycosylation Sites	Numbers
CH-1a	N34, N44, N5	3
CH-1R	N34, N44, N51	3
SD1612-1	N30, N34, N44, N51	4
JXA1	N30, N35, N44, N5	4
WUH4	N30, N44, N51	3
HUB1	N30, N35, N44, N5	4
HUN4	N30, N35, N44, N51	4
TJ	N30, N35, N44, N51	4
GD	N30, N35, N44, N51	4
BJ-4	N30, N33, N44, N51	4
SD1-100	N30, N33, N44, N51	4
RespPRRS MLV	N30, N33, N44, N51	4
DY	N30, N33, N44, N51	4
VR2332	N33, N44, N51	3
FJSD	N30, N33, N44, N51	4
QYYZ	N30, N33, N44, N51	4
GM2	N34, N44, N51	3
IA/2014/NADC34	N32, N33, N44, N51, N57	5
HLJTZJ921-2011	N32, N33, N44, N51, N57	5
FJ0908	N32, N33, N44, N51	4
LNWK130	N32, N33, N44, N51	4
JS2021NADC34	N32, N33, N44, N51	4
SD	N32, N33, N43, N50	4
HLJ-80	N32, N33, N43, N50	4
SWU/CD1/2018	N32, N33, N43, N50	4
XJ-Z5	N33, N43, N50, N56	4
GSFEI2-2023	N32, N33, N43, N50	4
XJ-2020	N32, N33, N43, N50, N58	5
NADC30	N32, N43, N50	3
HNjz15	N34, N44, N51	3
HENAN-HEB	N32, N44, N51	3
CHsx1401	N34, N44, N51	3
JL580	N34, N44, N51	3
Minnesota7	N30, N34, N44, N51	4
HeB2023092	N30, N33, N44, N51	4

**Table 4:** Recombinant events of HeB2023092 strain identified by RDP5.

No.	Breakpoint Positions		Minor Strains	Major Strains	Detection Methods						
	Begin	End			R	G	B	M	C	S	T
1	12,152	13,961	JS2021NADC34	NADC30	+	+	+	+	+	+	+
2	1	1508	JXA1	NADC30	+	+	+	+	+	+	+
3	5280	6568	JXA1	NADC30	+	+	+	+	+	+	+
4	6589	8076	JXA1	NADC30	+	+	+	+	+	+	+

## Data Availability

The original contributions presented in this study are included in the article/Supplementary Materials. Further inquiries can be directed to the corresponding author.

## Supplementary Materials

The following supporting information can be downloaded at: https://www.mdpi.com/article/10.3390/vetsci12100983/s1, Figure S1: Identification of the PCR product of Nsp2 from the strain HeB2023092. File S1: HeB2023092 genome sequence.
