# Supplementary material for: Molecular Characterization of a Recombinant NADC30-like PRRSV Strain with a Novel Gene Deletion Pattern in Nsp2 Gene
Source: Vet Sci. 2025 Oct 13;12(10):983. doi: 10.3390/vetsci12100983 (PMC12567703; doi:10.3390/vetsci12100983)
Supplement: Supplementary file 1 [file vetsci-12-00983-s001.zip › vetsci-3910082-initial submission supplementary.pdf]

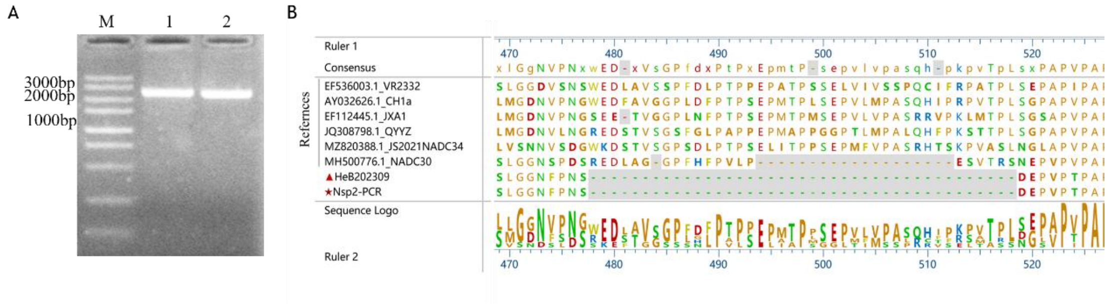

Figure S1. Identification of the PCR product of Nsp2 from the strain HeB2023092. (A) Identification of Nsp2 using PCR. Lane 1 displays Nsp2 from an NADC30-like strain, while Lane 2 shows Nsp2 from HeB2023092. (B) Sequence alignment of amplified Nsp2 amino acids. The Nsp2 PCR sequencing from HeB2023092 is highlighted in red (★).
