# Supplementary material for: Molecular Characterization of a Recombinant NADC30-like PRRSV Strain with a Novel Gene Deletion Pattern in Nsp2 Gene
Source: Vet Sci. 2025 Oct 13;12(10):983. doi: 10.3390/vetsci12100983 (PMC12567703; doi:10.3390/vetsci12100983)
Supplement: Supplementary file 1 [file vetsci-12-00983-s001.zip › vetsci-3910082-supplementary.pdf]

File S1. HeB2023092 genome sequence

ATGACGTATAGGTGTTGGCTCTATGCCACGACATTTGTAG
TGTCAGGAGCTGTGACCACTGGCACAGCCCAAAACTTGC
TGCACGGGAACACCCTCCCGTGACAGTTCTCTTCAGGGG
ATTAGGGGTCTGTCCCTAACACCTTGCTTCCGGAGTTGCA
CTGCTTTACGGTCTCTCCACACCTTTAACCATGTCTGGGA
TGCTTGATCGGTGCACGTGCACCCCCAATGCCAGGGTGTT
TATGGCGGAGGGCCAGGTCTACTGCACACGATGTCTCAG
TGCACGGTCCCTCCTTCCTCTGAATCTCCAAGTGTCTGAG
CTTGGGGTGCTGGGTCTATTTTATAGGCCCGAAGAGCCTC
TCCGGTGGAAGTTGCCATATCCATACCCCACTGTCGAGT
GCTCCCCCGCCGGGGCCTGTTGGCTTTCTGCGATCTTTCC
GATTGCACGAATGACTAGTGGAAACCTGAACYTTCAACA
AAGACTGGTGCGGGTTGCGGCTGAAATCTACAGGGCCGG
CCAACTCACCCCTGCAGTCCTAAAGGCTCTACAAGTCTA
TGAACGGGGTGGTCGTTGGTACCCCATTGTCGGGCCCGTC
CCTGGGGTGGCCGTTTACGCCAACTCCCTGCACGTGAGT
GATAAACCTTTTCCGGGAGCTACTTATGTATTGACCAATT
TGCCGCTCCCGCAGAGGCCCAAACCTGAGGACTTTTGCC
CTTTTGAGTGTGCCATGGCTGATGTCTATGATATTGGTCG
TGACGCCGTCATGTACGTGGCCGGAGGAAAGGTTTCTTG
GGCCCCTCGTGGTGGGAATGAGGTGAAATTTGAACTTGT
CCCCAAGGATCTTGAGTTGGTTGCGAGCCGGCTCCACAC
CTCCTTTCCGCCTCATCACGTAGTGGACATGTCCGATTTT
ACCTTCGTGACCCCCGGGAGTGGTGTCTCTATGCGGGTTG
AGTACCAACATGGCTGCCTCCCAGCTGACACCGTCCCTG
AAGGAAACTGCTGGTGGCGCTTGTTTGACTCGCTCCTACC
TGAGGTTCAGTACAGAGAGATTCGCTATGCTAATCAGTTT
GGCTATCAAACCAAGCATGGTGTCTCTGGCAAATACCTA
CAGCGGAGGCTGCAGATTAATGGTCTTCGAGCAGTGGTC
GACACACACGGACCAATTGTCATACAGTATTTCTCTGTTA
AGGAGAGCTGGATTCGTCACCTGAAGTTGGTGGAAGAAC
CCAGCCTCCCTGGATTTAAGGACCTCCTCAGGATCAGGG
TCGAGCCCAATACGTCACCACTGGTTGAAAAGGATGAGA
AGATCTTTCGGTTTGGCAGCCATAAGTGGTACGGTGCTGG
AAAGAGAGCAARAAAAACGCGCCCTAGTGCTACTACTG
TGGTCGCTCGTCAAGCTTCGTCTGCTCATGAAACCCGGCA
TGCCACGAAGCACGAGGGTGCCGGCGCTAACAAGACTA
AGCACCCCGATCGCTACTCTCCGCCTGCCGAAGGGAACT
GTGGCTGGCACTGTCTCTCTGCAATCGCCAACCGGATGTT
GCATTCCAAATTTGAAACCACCCTTCCCGAAAGAGTGAG
GCCCCCAAATGACTGGGCAACTGATGAAGATCTTGCCAA
TGCTATCCAAACCCTCAGGCTTCCTGCGGCTTTGGACAGG
AATGGTGCTTGTAAAGGCGCCAAGTATGTTCTTAAGCTG
GAAGGCGAGCACTGGACTGTCTCAGTTACCCCTGGGATG
CCTCCTTCTTTGCTCCCTCTTGAATGTGTTCAGGGTTGCTG
TGAGCACAAAGGTGATCTCAACCCCCCAGGTACAGTCGA
GGTCTCCGGGTTTGACCCCGCCTGCCTTGACTGGTTGGCC
GAAGTGATGCATCTGCCTAGTAGTGCTATCCCTGCCGCCC

TATCCGAGATGTCCAGCAGCTTTGGTCGTACGACCCCTCC
GGTTCCTGTTGTGTGGACCGTTTCACAGTTCTTTGCTCACC
GCAGCGGAGGGGATCACTCTGATCAAGCATGCTTGGAGA
AAATTGTCAATCTTTGTCAGCTGCTTGAAAGCTGCTGCTG
CTCTCAGAACAAAGCCAACCCGGTCACCCCGGGGGAGG
TGAGAGGAAAAATTGATCAGTACCTCCGTGGTGCAGTAA
ATCTTGAAGAATGTTTGGCTAGGCTTGAGAAAGCCCGCC
CGCCCAGTGTGTTGGACACCTCCTTTGATTGGAATTTTGT
GCTACCTGGAGTTGAAGTGGCCGCTCAAGTAGCGGAACT
GCCCCTCACTAACCAGTGTCGCGCTCCTATCACTGTTGCG
GCCCAAATGTCTTCGCCGGGATTCCAGTCTCGAAAAGCG
GAGTCTGTCCGGAGTCTTCCAGAGGAAAGGCCCCTTCCC
GCCCCACGCAGGAAGATCAGGCCCAGGTGCGGAAGTTT
GGTTTCATTGGGCGGCAACTTTCCTAACTCGGATGAGCCT
GTGCCTACCCCCGCACCACGCAGGACCGTGTCCCGGCCT
AAGCTGCCATCGATGACGTCAACCCCTGTGCCTGCACCG
CGGTATGGGCTTCAGCAGGTGGGGGGAATAAATTTGGCG
GTAGGAACTCTTGCGTGCCAGGGCGAGCTCCTCGATTTGT
CTGCATCTTCGCAAACTGAATACGAGGCTTCCCCTTTGGC
ATTGCCGCATAGTGAGGTCGCCCTGAAGGTGGGGGGACG
AGATGCTGTGGAAGTTCTGAGCGAAGCCTCGGGCGAGTC
GGACGGTATCAGACTGACACCCGTGTCGTCAAGCAGCTC
CCTGTCAAGCGTTGAGATCACACGCCCAAAATACTCAGC
TCAGGCCATCATTGACACAGGCGGGCCCTGTTGTGGACA
CCTCCAAGAGGTAAAGGAGAAATATCTCAATGTGATGCG
TGAAGCATGTGATGCGACTAAACTTGATGACCCTGCCAC
ACTAGAGTGGCTTTCCCGCATGTGGGATAGAGTGGACAT
GCTAACCTGGCGCTGTACGTCCGCTTTTCAAGCACCTTTC
GTCTTGGCTGACAAGTTCAAGTTCCTCCCAAAGATGATA
CTTGAAACACCACCGCCCTACCCTTGCGGGTTCGTGATG
ATGCCCCGCACACCGGCACCTTCTATAGGTGTGGAGAGT
GACCTTACCGTCTGTTCAGTTGCCACTGAAGACGTTCCGC
GCCTTCTCGGAAAGGTAAGAGATGCTAGCAAGACGACC
GGCCAGGCGCCCTTTACGTCCTCTATAGATGAGCCAACT
GATGGCCAACCTGCTGGAGAGCCCCGGGCGCAGGCTCCT
CCCGCGGGTGCAGATGGTGTCGACTTAGTTTTAGACTCTG
GAGGACCGCCGGAGCTCGCTGACTTGTCGTCTCCAAACG
GTGCTGGCACAGATGGCGTGGGACCGTTACGTACGGTCA
GGAAAGAGGCTGAGAAGTACTTTGACCAGCTGAGCCGG
CGGGTTTTTGGTATCGTCTCCCATCTCCCTGTCTTCTTCTC
ACGCCTGTTCAGGCCCGATGGTCGTTATTCTCCGGGTGAT
TGGGGCTTTGCAGCTTTTACTTTATTGTGCCTCCTTCTGTG
TTACAGTTATCCTGCGTTTGGTGCCGCTCCCCTATTGGGT
GTATTTTCTGGGACTTCTCGGCGCGTTCGCATGGGGGTTT
TTGGCTGCTGGCTGGCTTTTGCTATTAGCTTGTTCAAGCCT
GTGTCCGACCCAGTCGGCACTGCTTGTGAGTTTGACTCGC
CAGAATGTAGAGACATCCTTCATTCTTTTGAGCTTCTGCA
ACCTTGGGACCCTGTTCGCAGCCTTGTGGTTGGCCCCGTC
GGTCTCTGTTTTGCCGTTCTTGGCAGGTTACTGGGCGGCG

CACGCTACGTCTGGTTGCTTCTGCTTAGGCTTGGCATCCTT
TCAGATTGTGTCCTGGCTGGAGCCTATGTGCTTTCGCAGG
GTAGGTGTAAAAGGTGTTGGGGATCTTGTGTGAGAACCG
CTCCAAGTGAGGTTCCCTTCAACGTGTTTCCCTTTACGCG
TGCGACCAGGTCATCGCTCATCGATCTGTGCGATCGATTT
TGCGCACCAAAGGGAATGGACCCTATTTTCCTCGCTACC
GGATGGCGCGGATGCTGGATTGGCCAAAGCCCCATTGAG
CAACCATCTGAGAAACCTATCGCGTATGCCCAATTGGAT
GAAAAGAAGATCACGGCTAAGACTGTGGTCACCCAACC
CTACGATCCCAATCAGGCTGTAAAGTGCTTACGGGTTTTG
CAGGCTGGTGGAGCGATGGTGGCGGAGGCTGTCCCAAA
AGTGATTAAGGTTTCTGCTATCCCATTTCGAGCCCCCTTTT
TTCCCACTGGAGTGAAAGTTGACCCCGATTGCAGAATTG
TGGTCGACCCTGATACCTTTACAACGGCCCTCCGGTCCGG
CTATTCTACCTCAAACCTCGTCCTTGGTGTTGGGGATTTC
GCCCAGCTAAATGGATTGAAAATTAGGCAGATCTCCAAG
TCCTCAGGGGGAGGCCCACACCTCGCAGCTGCCCTACAT
GTTGCTTGCTCGGTGGCACTGCACATGCTTGCCGGAATTT
TTGTGACCTCGGTTGGCACCTGTGGTACTGGTACCAACGA
CCCGTGGTGTACCAACCCGTTTGCTGTCCCTGGTTACGGA
CCCGGCTCCCTCTGCACGTCCAGGTTGTGCATCTCCCAGC
ATGGTCTTACGCTACCATTGACTGCGTTAGTGGCCGGGTT
CGGCATGCAGGAGCTTGCTTTGGTTGTTTTGATTTTTGTTT
CTATCGGTGGCTTGGCTCACCGGTTAAGTTGCAAGGCTGA
TGTGGTGTTTGTTTTACTTGCAATTGCCAGCTATGTTTGGG
AACCTCTCACCTGGTTGCTTTGTGTGTTTCCCTGTTTRTTG
CGCTGGTTTTCTTTGCACCCCCTCACCATCCTTTGGTTGGT
GTTTTTCTTGATTTCTGTGAATATACCCTCGGGGATCTTGG
CCGTGGTGTTACTGGTTTCTCTCTGGCTCCTAGGTCGTTAT
ACTAACATTGCGGGTCTCGTTACTCCCTATGACATTCATC
ATTACACCAGTGGCCCGCGCGGTGTTGCCGCCTTGGCCA
CTGCACCAGACGGAACCTACTTGGCTGCCGTCCGCCGAG
CTGCGCTGACTGGTCGTACCATGCTGTTTACCCCGTCCCA
GCTCGGGTCTCTCCTTGAGGGCGCTTTTAGAACTCAAAAG
CCCTCACTGAACACCGTCAATGTGGTCGGGTCCTCCATG
GGCTCTGGCGGAGTGTTCTCTATTGACGGGAAAATTAAG
TGCGTGACTGCCGCACATGTCCTTACAGGCAACTCAGCT
AGGGTTTCCGGGGTTGGCTTCAATCAAATGCTCGACTTTG
ATGTAAGAGGAGACTTCGCCATAGCTGATTGCCCGAATT
GGCAAGGGGTTGCTCCCAAGGCCCGGTTCTGCGAGGATG
GGTGGACTGGTCGCGCCTATTGGCTGACATCCTCTGGCGT
CGAGCCCGGTGTTATTGGGAATGGGTTCGCCTTCTGCTTC
ACCGCGTGTGGCGATTCTGGGTCCCCAGTGATCACCGAA
GCCGGTGAGATTGTCGGCGTTCACACAGGATCAAACAAA
CAAGGAGGAGGCATTGTCACGCGCCCCTCAGGCCAGTTC
TGTAATGTGAAGCCCATCAAGCTGAGCGAGTTGAGTGAA
TTCTTCGCTGGACCTAGGGTCCCGCTCGGCGATGTGAAA
ATCAGCAATCACATAATTAAAGACATACACGAGGTGCCC
TCAGATCTGTGTGCCCTACTAGCAGCCAAACCCGAACTG

GAAGGAGGCCTTTCCACAGTTCAACTCCTGTGTGTGTTCT
TCCTCCTGTGGAGAATGATGGGGCATGCCTGGACGCCCT
TGGTTGCCGTGGGGTTTTTCATCTTGAATGAGATTCTCCC
AGCTGTTCTGGTCCGGAGTGTTTTCTCCTTCGGGATGTTTG
TGCTGTCTTGGCTTACGCCATGGTCTGCGCAAGTCTTAAT
GATCAGGCTTCTGACAGCAGCCCTTAATAGAAATAGATT
GTCTCTCGGGTTTTTCAGCCTTGGTGCAGTAACCGGTTTTT
TTGCAGATCTTGCGGTAACTCAAGGGCACCCGTTGCAAG
TAGTGATGAACTTAAGCACCTATGCCTTTCTGCCTCGGAC
AATGGTTGTGACCTCGCCAGTCCCAGTGATCGCGTGTGGT
GTTGTGCACCTCCTCGCCATAATTCTATACTTGTTTAAATG
TCGCAGCCTTCATAGTGTCCTTGTTGGCGATGGTGTGTTCT
CTTCAGCTTTCTTCTTGCGGTACTTTGCCGAGGGGAAGTT
GAGGGAAGGGGTGTCACAATCCTGCGGGATGAGCCACG
AGTCGTTGACTGGTGCTCTCGCCATGAGACTCACTGACG
AGGACTTGGACTTTCTTACGAAATGGACTGACTTCAAGT
GCTTTGTTTCTGCGTCCAACATGAGGAATGCAGCGGGCC
AATTCATCGAGGCTGCTTACGCAAAAGCACTAAGAGTTG
AACTTGCCCAGTTGGTACAGGTTGACAAAGTCCGAGGCA
CCTTGGCCAAACTTGAGGCTTTTGCTGATACTGTTGCGCC
ACAAGTCTCGCCCGGTGACATCGTTGTTGCCCTCGGCCAC
ACGCCTGTTGGCAGCATCTTCGACCTAAGGGTTGGTAGC
ACCAAGCACACCCTCCAAGCCATTGAAACTAGGGTCATT
GCCGGGTCCAAAATGACTGTAGCGCGTGTCGTTGACCCG
ACCCCAGCACCCCCGCCCGTACCTGTGCCTATTCCTCTCC
CACCGAAAGTTCTGGAGAACGGTCCTAACGCCTGGGGGG
ATGAGGATCGCTTGAACAAGAAGAAGAGGCGCAGGATG
GAAGCCGTCGGCATCTTTGTCATGGACGGGAGAAAGTAC
CAGAAATTTTGGGACAAGAATACCGGTGATGTGTTTTAT
GAGGAGGTCCACAACAGCACAGATGAGTGGGAATGCCT
TAGGGCTGAAAACCCTGCCGACTTTGACCCCGAGACAGG
GGTTCCGTGTGGGCACATCGTCATTGAAGGCAGGGTTTAT
AATGCCTTCACCTCCCCATCTGGTAAGAAATTCTTGGTCC
CCGCTGACCCCGAGAACAGAAGAGCCCAATGGGAAGCC
GCCAAGCTTTCCGTGGAACAAGCCCTTGGTATGATGAAC
GTCGACGGCGAACTGACTGCCAAAGAAGTGGAGAAACT
GAAAAAGATAATTGAGAAACTCCAGGGCCTGACTAAGG
AGCAGTGTTTAAACTGCTAGCCGCCAGCGGCTTGACCCG
CTGTGGTCGCGGCGGCTTAGTTGTTACTGAGACAGCGGT
GAAGATAGTTAAATTCCACAACCGTACCTTTACCCTAGG
ACCTGTGAATTTAAAGGTGGCCAGTGAGGTTGAGCTTAA
AGACGCGATTGGGCACAACCAACACCCGGTAGCCAGGC
CGGCTGATGGTGGTGTTGTGCTCCTGCGCTCTGCAGTTCC
TTCGCTTATAGACGTCTTGATCTCCGGCGCCGATGCATCT
CCTAAGTTACTCGCCTGCCACGGGCCGGGAAACACCGGG
ATTAATGGCGCGCTTTGGGATTTTGAGGCCGAGGCTACTA
AAGAGGAGATTGCGCTCAGTGCGCAGATAATACAGGCTT
GTGACATTAGACGCGGCGATGCACCTAACATCGGCCTCC
CTTACAAGCTGCACCCTGTCAGAGGCAACCCTGAACGAG

TGAAAGGGGTTCTAAAGAACACGAGGTTTGGGGACATAC
CTTACAAGACCCCCAGTGATACTGGGAGCCCAGTGCATG
CGGCCGCTTGTCTTACGTCCAATGCCACCCCGGTGACTGA
CGGGCGCTCTATCTTGGCCACGACCATGCCCTCCGGGTTT
GAGTTATATGTGCCGACTATCCCTGCGTCTGTCCTTGATT
ATCTTGATTCCAGGCCAGACTGCCCCAAACAGCTTACGG
AGCATGGATGTGAAGATGCCGCACTCAGAGACCTTTCCA
AATATGACTTGTCCACCCAAGGGTTTGTTTTGCCTGGGGT
CCTCCGTCTTGTACGGAAGTATTTGTTTGCTCACGTTGGC
AAGTGCCCACCCATTCATCGACCCTCTACCTATCCAGCC
AAGAATTCTATGGCTGGAATAAATGGGAACAGGTTCCCA
ACCAAAGACATTCAGAGCGTCCCTGACATCGACGTCCTA
TGTGCACAGGCTGTGCGTGAGAACTGGCAGACTGTTACC
CCTTGCACCCTCAAGAAGCAGTATTGCGGTAAGAAGAAA
ACCAGGACCATACTGGGTACCAATAATTTTGTTGCGCTG
GCACACCGGGCAGCACTGAGTGGTATCACCCAGGGTTTC
ATGAAAAAAGCATTCAACTCTCCTATTGCCCTTGGTAAA
AACAAATTTAAGGAGCTGCAAACTCCAGTCCTAGGCAGG
TGCCTTGAGGCTGATCTTGCGTCTTGCGATCGGTCCACCC
CCGCAATTGTTCGCTGGTTTGCCGCCCACCTCCTTTATGA
ACTTGCTTGTGCTGAGGATTACCTACCGTCGTACGTGCTG
AACTGCTGCCATGATCTGCTGGTCACGCAGTCCGGTGCA
GTGACTAAAAGGGGTGGCCTGTCATCTGGTGACCCGATC
ACATCTGTGTCTAACACCATTTACAGTTTGGTGATTTATG
CGCAGCATATGGTGCTCAGTTACTTCAAAAGCGGTCATC
CACACGGTCTCCTGTTCCTCCAGGATCAGCTCAAGTTTGA
GGACATGCTAAAGGTCCAACCACTGATTGTCTACTCGGA
TGATCTTGTGCTGTATGCCGAATCCCCCTCTATGCCTAATT
ATCACTGGTGGGTTGAGCATCTTAATCTGATGCTAGGGTT
CCAAACAGACCCAAAGAAGACAGCTATCACTGACTCGC
CGTCTTTTCTAGGCTGCAGGATAATCAACGGACGTCAGCT
AGTTCCAAACCGTGACAGGATTCTTGCAGCTCTTGCCTAC
CACATGAAGGCGAGTAATGTTTCTGAGTACTACGCCTCT
GCGGCTGCAATACTCATGGACAGCTGTGCATGTCTGGAG
TATGACCCTGATTGGTTTGAAGAGCTTGTGGTTGGTATGG
CGCAGTGCGCCCGCAAGGACGGTTACAGTTTTCCCGGCC
CGCCATTCTTCCTATCCATGTGGAAAAACTCAGGTCTAA
TTATGAGGGAAAGAAGTCAAGGGTGTGTGGGTACTGTGG
AGCTTCAGCCCCGTACGCCACTTCCTGCGGTCTAGACGTT
TGTGTTTACCACACCCACTTTCACCAGCATTGCCCTGTCA
TAATCTGGTGTGGCCATCCAGCGGGTTCCGGGTCCTGTGA
CGAGTGTAAATCTCCTGTAGGCAAAGGTACGAGCCCCTT
GGATGAGGTCTTGAGGCAAGTTCCGTACAAACCTCCACG
GACCGTCCTTATGCACGTTGAGCAGGGCCTCACCCCCCTT
GACCCAGGCCGATATCAGACCCGCCGTGGGTTAGTTGCC
GTCAGACGTGGGATCAGGGGAAATGAAGTTGATTTACCA
GATGGTGATTATGCCAGCACCGCCTTACTTCCAACTTGTA
AGGAGATCAACATGGTTGCCGTTGCTTCCAATGTGTTGCG
CAGCAGATTCATCATCGGCCCGCCCGGTGCTGGGAAGAC

ATATTGGCTCCTTCAACAGGTTCAGGATGGTGATGTCATT
TACACACCGACCCATCAGACCATGCTAGACATGATTAAA
GCTTTGGGAACGTGTCGGTTTAATGTCCCGGCAGGCACA
ACGTTGCAATTTCCCGCTCCTTCCCGTACCGGCCCGTGGG
TTCGAATCCTGGCCGGCGGGTGGTGCCCTGGAAAAAACT
CCTTCCTGGACGAAGCGGCGTATTGCAATCATCTTGATGT
CTTGAGACTCCTTAGTAAAACCACTCTTACCTGTTTGGGA
GACTTCAAACAACTCCACCCGGTGGGTTTTGATTCACACT
GTTATGTTTTTGACATCATGCCTCAGACTCAACTGAAAAC
CATCTGGAGATTTGGGCAGAACATCTGTGAGGCCATTCA
GCCTGATTACAGAGACAAGCTAGTGTCTATGGTTAACGC
AACTCGTGTAACGTACGTGGAAAAACCTGTCAAATACGG
GCAAGTCCTCACCCCTTACCATAGGGACCGAGATGACAC
TGCTATTACCATTGACTCTAGTCAAGGCGCCACATTTGAT
GTGGTCACACTGCACCTGCCCACAGAAGATTCACTCAAT
AAACAAAGAGCCCTTGTTGCCATCACCAGGGCAAGACAT
GCCATTTTTGTGTATGACCCACATAAGCAATTGCAGAGC
CTATTTGATCTTCCTGCAAAGAGCACGCCCGTCAATCTGG
CCGTGTTCCGTGATGGGCAACTGATCGTGCTAGACAGAA
ACAATAAAGAGTGCACGGTTGCCCAAGCTTTGGGCAATG
GTGACAAATTTAGGGCTACAGATAAGCGCGTTGTAGATT
CTCTCCGCGCCATTTGTGCAGATCTGGAAGGGTCGAGCTC
CCCGCTCCCCAAGGTCGCACATAACTTAGGATTTTATTTT
TCACCGGATTTGACACAGTTTGCTAGGCTCCCGGCAGAA
CTTGCACCTCACTGGCCAGTGGTGACAACCCAGAACAAT
GAAAAGTGGCCAGACCGGTTAGTTGCTAGCCTTCGCCCC
ATCCATAAGTATAGCCGCGCGTGCATTGGTGCCGGCTAC
ATGGTGGGCCCCTCAGTGTTTCTGGGCACCCCCGGGGTA
GCATCATACTATCTTACAAAGTTCATTAGAGGAGAAGCC
CAAATGCTTCCAGAGACGGTCTTCAGCACCGGTCGAATT
GAGGTGGATTGCCGTGAGTATCTCGATGACCGGGAACGA
GAGGTTGCTGAGTCTCTCCCCCATGCTTTCATTGGCGACG
TCAAGGGCACCACCGTCGGAGGATGTCATCACGTCACCT
CCAAATATCTCCCGCGCTTCCTTCCCAAGGAATCAGTCGC
GGTAGTCGGGGTTTCAAGCCCCGGGAAAGCCGCAAAAG
CAGTTTGCACATTGACGGATGTGTATCTACCAGATCTTGA
AGCTTACCTTCACCCGGAGACTCTGTCCAAGTGTTGGAA
AATGATGTTGGACTTCAAGGAAGTCCGACTGATGGTCTG
GAAGGACAAGACGGCCTATTTTCAACTTGAAGGCCGCCA
TTTCACCTGGTACCATCTTGCAAGCTACGCATCGTACATC
CGAGTTCCTGTTAACTCTACTGTGTATCTGGACCCTTGCA
TGGGCCCCGCCTTTTGCAACAGGAGAGTTGTTGGGTCCA
CTCATTGGGGAGCTGACCTCGCAGTCACCCCTTACGATTA
CGGTGCTAAAATCGTCCTGTCTAGTGCATACCATGGTGA
GATGCCTCCCGGATACAAAATTCTGGCGTGCGCGGAGTT
CTCGTTTGACGATCCAGTGAGGTACAAACACACCTGGGG
GTTTGAGTCGGATACAGCGTATCTGTACGAGCTTACCGG
GGACGGTGAAGATTGGGGAGATTACAATGAAGCATTTCG
TGCGCGCCAGAAAGGGAAAATTTACAAGGCCACTGCTA

CCAGCTTGAGGTTTCATTTTCCCCCGGGTCCCATCATTGA
ACCAACTTTAGGCTCGAACTGAAATGAAATGGGGGCTTT
GCAGAGCATCTTTGACAAAATCGGTCAACTTTTTGTGGAC
GCTTTCACGGAGTTCTTGGTTTCTATTGTTGACATTGTCAT
ATTTCTGGCCATTTTGTTCGGCTTCACAGTCGTCGGTTGGT
TGGTTGTCTTTTGCATCAGATTGGTTTGCTCCACGATACTC
CGTGCGCGCCCTGCCGTTTACCCTGAGCAGTTACAGAAG
GTCCTATGAGGTTTTCCTCCCCCTGTGCCAGACGGACACC
CCCATCTGGGGAACCAAGCACCCCCTAGGAATGTCTTGG
CACCACAAGGTTTCAACCCTGATTGATGAAATGGTGTCG
CGTCGAATGTACCGCACCATGGAACATGCAGGACAGGCT
GCCTGGAAACAGGTGGTGAGCGAGGCTACTCTGTCTCGC
ATTAGCAGCCTGGATGTAGTGGCCCATTTCCAGCATCTCG
CCGCCATTGAGGCCGAGACCTGCAAATATTTGGCCTCCC
GGCTGCCCATGCTACACAATCTACGCATGACAGGGTCGA
ATGTAACCATAGTGTATAACAGCACTTCGAATCGGGTTTC
CGCTATCTTCCCGACCCCCGGTTCCCGGCCAAAACTTCAT
GATTTTCAGCAATGGCTAATAGCTGTACATTCCTCTATAT
TCTCTTCTGTTGCAGCTTCTTGCACTCTCTTTGTTGTGTTGT
GGTTGCGGATTCCAACGCTACGTTCTGTTTTTGGTTTCCGC
TGGTTAGGGGCAATTTTTCCTTCGAGCTCCTTGTAAATTA
CACGGTGTGCCAGCCTTGCCTTACCCGACAAGCAGCCGC
CCAGATTTTTCAACCTGGCAGATCCCTTTGGTGYAAGATT
GGGCATGATCGATGTGAGGAGAGCGACCATGATGAGCT
AGGGTTTGTGGTACCGCCTGGTCTTTCCAGCGAAGGTCAC
TTGACCAGTGCTTATGCCTGGTTGGCGTTCCTGTCCTTCA
GTTATGCAGCCCAGTTTCATCCCGAGATATTTGGGATCGG
GAATGTGAGCACCGTTTATGTCGACAAACCCAATCAACT
CATTTGCGCTGTTCATGATGGGCAGAACGCCACCTTGTCC
CGCTATGATAACATCACAGCCGTGTACCAGACCTACTAT
CAACACCAAGTTGACGGAGGCAATTGGTTTCACCTAGAA
TGGCTGCGTCCTTTCTTTTCCTCTTGGTTGGTTTTGAACGTT
TCATGGTTTCTCAGGCGTTCGCCTGCAAACCGTGCTTCAG
TTCGAGTCTTTCAGATATCAAAACCAACACCACCAGCGC
AGCAAGTTCCGTTGTCCTCCAAGACATCAGCTGTCTTAGG
CGTAACAACCCGTCCTCTGCGGCGTTTCGCAAAATCCCTC
AGTGCCGCACGGCGATAGGGACACCCGTCTACATTACCA
TCACAGCCAACGTGACGGATGAGAATTATTTGCATTCTTC
CGACCTTCTCATGCTCTCTTCTTGCCTTTTCTATGCTTCTG
AGATGAGCGAAAAAGGGTTCAAAGTGATATTTGGCAATG
TGTCAGGCATTGTGGCCGTGTGTGTCAACTTTACCAGCTA
TGTTCAACATGTCAAGGAGTTCACCCAGCGATCCTTAGTG
GTTGACCATGTGCGGCTGCTTCATTTCATGACACCTGAAA
CCATGCGATGGGCGACCGTTTTAGCCTGTCTTTTTGCCAT
TCTGCTGGCGATTTGAATGTTCAGGTATGTTGGGGAAATG
CTTGACCGCGGGCTGCTGCTCGCAATCGCTTTTTTTGTGGT
GTATCGTGCCGTTCTGTTTTGTTGTGCTCGTCAACGCCAG
CAACAGCAGCAGCTCCCACTTACAGTTGATTTATAACCT
GACGATATGTGAGCTGAATGGCACGGATTGGTTAAACAA

CAGATTTTCTTGGGCAGTGGAGACATTCGTCATCTTTCCT
GTGTTGACTCATATCGTCTCTTACGGTGCCCTCACCACTA
GCCATTTTCTTGACATGGTCGGCCTGATCACTGTGTCCAC
CGCCGGTTATTGTCACGGGCGGTATGTGCTGAGTAGCATC
TACGCTGTCTGTGCCCTAGCTGCGTTGGTTTGCTTCACCAT
TAGGTTGGTAAAAAATTGCATGTCCTGGCGCTACTCATGC
ACCAGATACACCAATTTTCTTCTGGACACTAAGGGTAAA
CTGTACCGTTGGCGGTCACCCATTATCATAGAGAAAGGG
GGCAAGGTTGATGTGGGGGGTCATTTCATCGACCTCAAG
AGAGTTGTGCTTGATGGTTCCGCGGCAACACCTGTAACC
AAGATTTCAGCGGAACAATGGTGTCGTCCATAGACGACT
TCTGCAATGACAGCACGGCTGTACAGAAGGTGTTATTGG
CGTTTTCCATCACCTACACACCAATAATGATATATGCCTT
AAAGGTAAGTCATGGTCGACTGCTGGGGCTCTTGCACCT
CGTAATTTTTCTGAATTGTGCTTTTACTTTTGGGTATATGA
CTTTTGTTCATTTTCAAAGTACAAACAAGGTTGCACTCAC
CATGGGGGCTGTGGTCGCTCTCCTCTGGGGGACCTATTCA
GCCATTGAAACCTGGAGATTCATCACCTCCAGGTGCCGG
TTGTGCTTGCTAGGCCGCAAGTACATTCTGGCCCCTACCC
ACCACGTTGAAAGTGCCGCAGGCTTCCATCCGATAACGG
CAAGTGATAACCACGCATTTGTCGTCCGGCGTCCCGGCT
CCACTACGGTTAACGGCACACTGGTGCCCGGGTTGAAGA
GCCTCGTGTTGGGTGGCAGAAGAGCTGTAAAACGAGGA
GTGGTGAACCTTGTTAAATATGCCAAGTAACAACGGCAG
ACAGCAAAACAAAAAGAAGGGGGATGGCCAGCCAGTC
AATCAGCTGTGCCAGATGTTGGGTCAGATTATCGCCCAA
CAGCGCCAGTCTAGAGGCAAGGGACCGGGAAAGAAGAA
TAGGAGTAGAAACCCGGAGAAGCCCCATTTTCCTCTAGC
AACTGAAGATGACGTCAGACATCACTTTACCCCTAGTGA
GCGACAATTGTGTCTGTCGTCAATCCGGACTGCTTTTAAT
CAAGGCGCTGGAACTTGTACCCTGTCAGATTCAGGGAGA
TTAAGTTACACTGTGGAGTTCAGTTTGCCTACTCATCACA
CCGTGCGCCTGATTCGCGTCACAACATCACCCTCAGCAT
GATGAGCTGGCATTCTTGAGACATTCCGGTGTTTGAATTG
GGAGAATGAGTGGTGAATGGCACTGATTGATATTGTGCC
TCTAAGTCACCTATTCAATTAGGGCGGCCGTATGGGGGT
AACATTTAATTGGCGAAAACCATGCGGCCGAAATTAAAA
AAAAAAAAAAAAA
