# Supplementary figures and images for: Molecular Characterization of a Recombinant NADC30-like PRRSV Strain with a Novel Gene Deletion Pattern in Nsp2 Gene
Source: Vet Sci. 2025 Oct 13;12(10):983. doi: 10.3390/vetsci12100983 (PMC12567703; doi:10.3390/vetsci12100983)

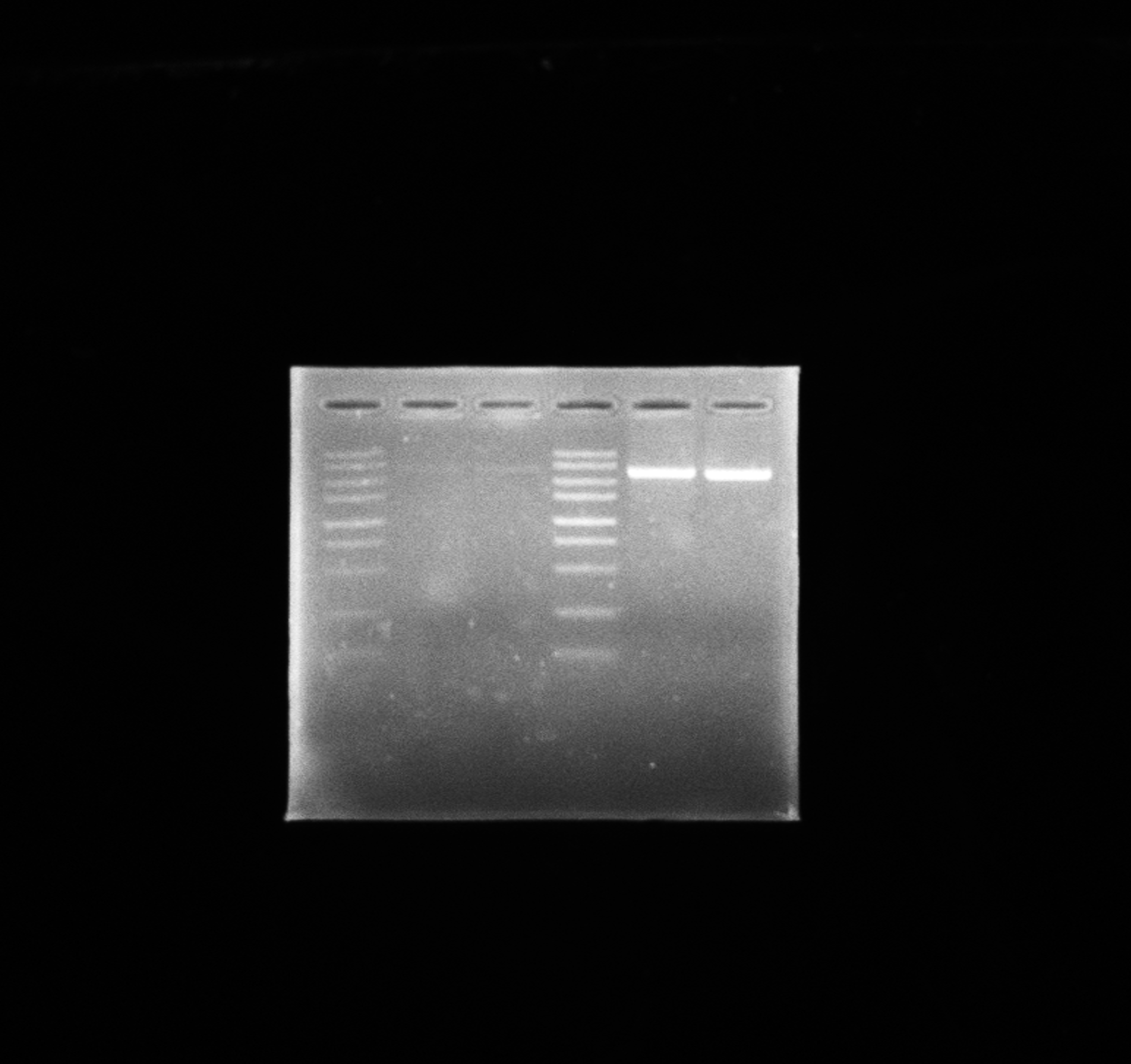

Supplement: Supplementary file 1 [file vetsci-12-00983-s001.zip › Identification of the PCR product of Nsp2 from the strain HeB2023092. (A) .tif]
